# Areas of Interest in Dental Education: A Bibliometric Analysis of the Last Decade

**DOI:** 10.7759/cureus.59589

**Published:** 2024-05-03

**Authors:** Eswar Kandaswamy

**Affiliations:** 1 Periodontics, School of Dentistry, Louisiana State University Health Sciences Center, New Orleans, USA

**Keywords:** dental hygiene education, scientometrics, trends, dental education, bibliometric analysis

## Abstract

This study aimed to perform a comprehensive bibliometric analysis of journals focused on dental education (Journal of Dental Education and European Journal of Dental Education) from 2014 to 2023. An ISI Web of Science Search was performed in October 2023 with no filters for language or keywords. Published articles between 2014 and 2018, 2019 and 2023, and 2014-2023, along with the top 100 cited articles published within this period were exported as txt files. Keyword and title word network maps and occurrences were generated using VOS Viewer software. Author-affiliated countries with the most publications were tabulated from the Web of Science. Dental education and dental students and education were consistently in the top six keywords and title word occurrences in all periods and top 100 cited articles. Similar trends were observed for keyword and title word network maps with an emphasis on dental education and students. However, the 2019-2023 period saw the emergence of coronavirus disease 2019, three-dimensional printing, virtual reality, and education technology, with the earlier period (2014-2018) showing clusters around students, perceptions, dental hygiene education, and assessment. The United States ranked top of the list for most published author-affiliated countries, with England, Canada, Australia, and Saudi Arabia in the top six for all periods analyzed. In conclusion, within the limitations of this study, areas of interest in dental education journals in the last decade were identified along with the countries with most publications.

## Introduction and background

Dental education is an important component of educating and training future dentists and dental specialists. In recent times, several challenges in the form of coronavirus disease 2019 (COVID-19) [1], educational needs and learning styles of modern-day students [2], as well as significant shifts in clinical dentistry and the evolution of concepts [3] have led to an increase in the importance and volume of dental education research. This can be evidenced by the Journal Citation Reports impact factors of dental education journals (such as the Journal of Dental Education and European Journal of Dental Education) increasing in recent times to their highest levels [4]. As the field of dentistry has evolved, so have the teaching methods and concepts in the field of dental education [5]. These trends are closely reflected in the research that is published in dental education. One validated method to assess trends in niche areas of research is through bibliometric analysis.

The bibliometric analysis involves the systematic analysis of the current status as well as trends of published literature in a particular field, topic, or journal [6,7]. Such analysis helps inform the reader of research patterns and the countries and authors involved in research publications in a particular domain [8]. Additionally, keywords and titles that are used to inform the reader of the scope of a particular paper can be longitudinally analyzed to report the trends of publication to identify what is of interest in a particular field. In the field of dentistry, there has been a recent interest in bibliometric analysis in various fields of clinical dentistry [9-13]. In dental education, previously published bibliometric analysis focused on a specific time frame [14], a particular field [15,16], or highly cited articles [5]. However, a comprehensive analysis of all the articles published in dental education journals in the last decade has not been conducted. Hence, this study aimed to perform a bibliometric analysis of the Journal of Dental Education and the European Journal of Dental Education from 2014 to 2023. Specifically, network maps of keywords and titles were constructed and analyzed in consecutive five-year periods of 2014-2018 and 2019-2023 along with the top 100 cited articles within that period. Additionally, keyword occurrences, title word occurrences, and country affiliations of the published authors were analyzed between 2014 and 2018, 2019 and 2023, and 2014 and 2023 along with the top 100 cited articles within that period.

## Review

Methodology

The methods used in this study were adapted from a previously published protocol [17]. The study was exempt from institutional review board (IRB) approval (IRB# 6006) and classified as non-human research utilizing publicly available data. An ISI Web of Science search was conducted until October 2023. The search was focused on two journals: the Journal of Dental Education and the European Journal of Dental Education from 2014 to 2023 split by a five-year duration (2014-2018 and 2019-2023). The top 100 cited articles published during that period were identified. Language and keyword filters were not applied and the results were sorted by highest citations and exported as txt files using the full record and cited references option. This option was chosen as it allows for detailed analysis of citation maps concerning the articles published. Editorials, meeting letters, meeting abstracts, corrections, software reviews, and biographical articles were excluded.

For keyword analysis, VOS Viewer software [18] was utilized. The number of occurrences of keywords and title words was computed from VOS viewer software for the entire period (2014-2023), two time periods (2014-2018; 2019-2023), as well as the top 100 cited papers. Keyword network maps were generated for the two time periods (2014-2018; 2019-2023) and the top 100 cited papers. For the generation of keyword network maps, the full counting option for keyword and keyword plus options with at least 10 occurrences for each keyword (for the two time periods) and at least three keyword occurrences for the top 100 cited articles were included in the network maps. Additionally, for title words, at least five occurrences were included for the two periods and at least two occurrences for the top 100 cited articles. Each color in the keyword and title word network maps represents a specific cluster. The top six keywords and title words were tabulated.

The author-affiliated countries for the three time periods and the top 100 cited articles were analyzed from the Web of Science data and the top six for each period were tabulated.

Results

A total of 4,312 results were identified in the two journals. After applying time-specific filters (2014-2023), a total of 2,832 articles were identified. After the removal of editorials, meeting letters, meeting abstracts, corrections, software reviews, and biographical items, a total of 2,380 articles were finally included. From 2019 to 2023, a total of 1,437 articles were identified, and from 2014 to 2018, a total of 947 articles were identified.

Keyword occurrences revealed that dental education was the top of the keywords used for all periods and the top 100 cited articles. Notably, dental students and students ranked high in the list for the three time periods and the top 100 cited articles. Words such as curriculum and assessment were prevalent between 2014 and 2018, being replaced in the top six list by performance and dentistry between 2019 and 2023 (Table 1).

**Table 1:** Most used keywords in publications from 2014 to 2018, 2019 to 2023, and 2014 to 2023, and the top 100 cited articles during that period.

2014–2018		2019–2023		2014–2023		Top cited 100	
Keywords	Occurrences	Keywords	Occurrences	Keywords	Occurrences	Keywords	Occurrences
Dental education	652	Dental education	473	Dental education	1125	Dental education	42
Education	208	Education	306	Education	514	Dental students	29
Dental students	145	Dental students	214	Dental students	359	Stress	18
Curriculum	121	Students	164	Students	282	Dentistry	16
Students	118	Dentistry	141	Dentistry	222	Education	16
Assessment	104	Performance	93	Curriculum	207	Health	14

Title word occurrences revealed that dental students and dental education were top of the list for all periods analyzed. The period between 2014 and 2018 was dominated by words such as assessment and education, which were being replaced by COVID-19 and students between 2019 and 2023. Interestingly, the title word “stress” featured prominently in the top 100 cited list, with others being dental school, education, and assessment (Table 2).

**Table 2:** Top title words from publications during the years 2014 and 2018, 2019 and 2023, and 2014 and 2023, and the top 100 cited articles during that period.

2014–2018		2019–2023		2014–2023		Top cited 100	
Title words	Occurrences	Title words	Occurrences	Title words	Occurrences	Title words	Occurrences
Dental student	158	Dental student	179	Dental student	299	Dental education	22
Dental education	73	Dental education	95	Dental education	153	Dental student	14
Dentistry	69	Assessment	95	Assessment	140	Stress	12
Study	69	Student	90	Education	126	Dental school	7
Education	65	COVID-19	89	Dentistry	114	Education	6
Assessment	65	Education	87	Student	114	Assessment	6

**Figure 1:**
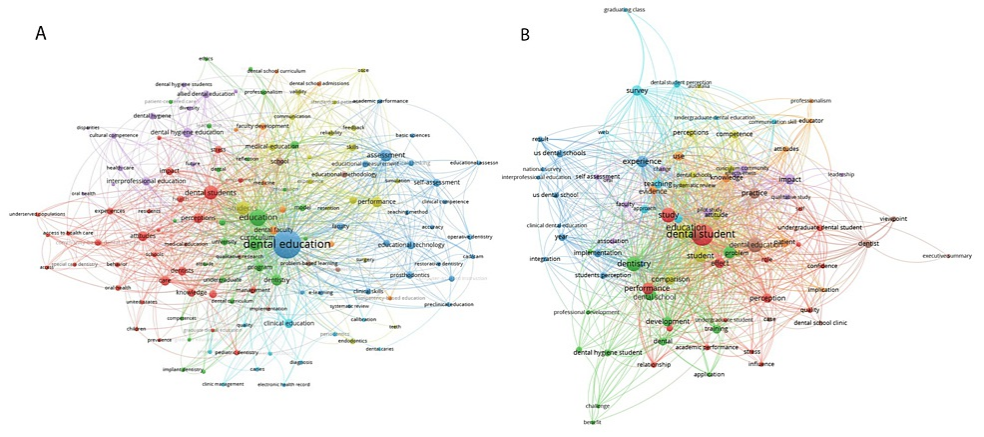
Keyword and title word analysis from 2014 to 2018. (A) (2014-2018) Keywords: Keyword cluster analysis revealed dental education (dark blue) connected to terms such as assessment, education technology, self-assessment, and prosthodontics; clinical education (light blue) connected to caries, diagnosis, and quality; dental students (red) connected to attitudes, knowledge, perceptions, and care; dental hygiene education (violet) connected to interprofessional education and allied dental education; education (green) connected to curriculum, dentistry, program, medical students; dental faculty (orange) connected to faculty development and medicine; students (yellow) connected to performance, endodontics, skills, and medical education; and school (brown) connected to education methodology, qualitative research, and problem-based learning. (B) (2014-2018) Title words: Title word cluster analysis revealed dental students (red) connected to study, performance, relationship, perception, and undergraduate student; experience (dark blue) connected to implementation, US dental school, national survey, student perception, and result; survey (light blue) connected to teaching, graduating class, and dental student perception; dental education (brown) connected to case, dental school clinic, practice, and dentist; dentistry (green) connected to development, training, dental, school, and dental hygiene student; patient (orange) connected to knowledge, use, evidence, and implication; impact (violet) connected to qualitative study, association, faculty, and self-assessment; and student (yellow) connected to education, comparison, attitude, competence, perceptions, and systematic review.

Keyword network maps revealed interesting differences between the two time periods and the top 100 cited articles. Keywords from 2014 to 2018 showed a large focus on dental education, which was, in turn, linked to keywords such as assessment and faculty. The word “students” was linked to medical education and performance, while the word dental student was linked to perceptions, attitudes, and knowledge. There was also a significant cluster around dental hygiene education noted in this period being linked to interprofessional education, allied dental education, cultural competence, and the future (Figure 1).

The title word analysis from 2014 to 2018 showed similar trends where a dental student was linked to study, performance, and perception. Other clusters were focused around dental education which was linked to practice and dentistry. A separate cluster around dentistry was linked to dental hygiene students, dental, development, and training (Figure 1).

**Figure 2:**
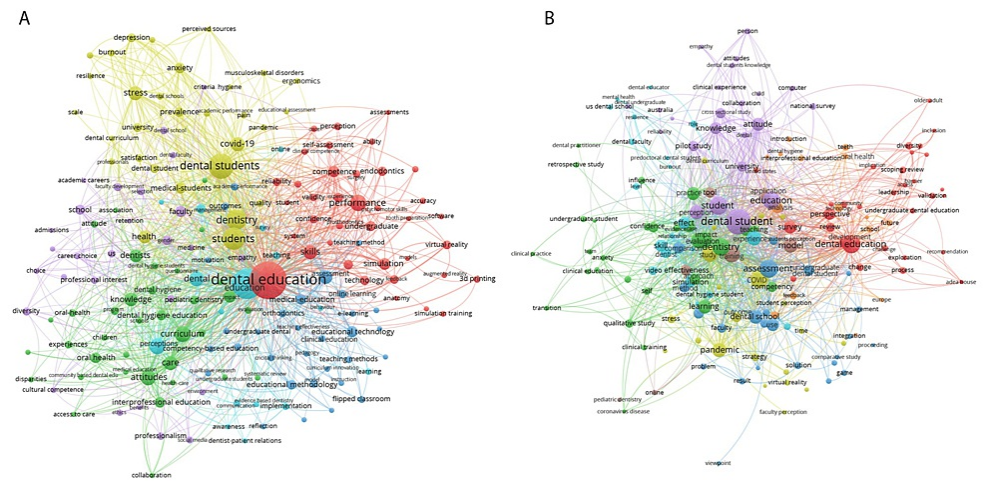
Keyword and title word network analysis from 2019 to 2023. (A) (2019-2023) Keywords: Keyword cluster analysis revealed dental education (red) related to skills, performance, competence simulation, technology, three-dimensional printing, and virtual reality; curriculum (green) related to knowledge, dentist, attitudes, oral health, and dental hygiene education; dental students (yellow) related to student, medical students, COVID-19, stress, prevalence, dentistry, health, burnout, and depression; medical education (dark blue) related to orthodontics, education methodology, educational technology, flipped classroom, and education technology; school (violet) related to career choice, professional interest, diversity, choice, faculty, and professionalism; education (light blue) related to perception, awareness, dental, implementation, dentist-patient relations, and management. (B) (2019-2023) Title words: Title word cluster analysis revealed dental student (violet) related to student, education, knowledge, attitude, and pilot study; dental education (red) related to perspective, change, survey, leadership, and review; pandemic (yellow) related to COVID-19, stress, virtual reality, outcome, study, and strategy; training (brown) related to effectiveness, model, and tool; technology (orange) related to analysis, teeth, oral health, interprofessional education, and future; skill (light blue) related to dentist, video, dental faculty, and US dental school; dentistry (green) related to evaluation, impact, confidence, learning, competency, and anxiety; school (orange) connected to future, analysis, teeth, and interprofessional education.

The keyword analysis from 2019 to 2023 also showed a large focus on dental education and was linked to performance, competence, and skills. Within that cluster, there was an emergence of words like simulation, technology, virtual reality, and three-dimensional (3D) printing. The word dental student was linked to dentistry, students, COVID-19, stress, and anxiety. In addition, there was a focus and cluster related to faculty, career choice, professional interest, and diversity. Greater emphasis was on attitudes, curriculum, knowledge, educational methodology, and interprofessional methodology. Furthermore, there was a cluster around education technology, education methodology, and e-learning. The title word analysis from 2019 to 2023 also showed similar trends with a large focus on dental students, education, assessment, knowledge, attitude, pandemic, and survey (Figure 2).

**Figure 3:**
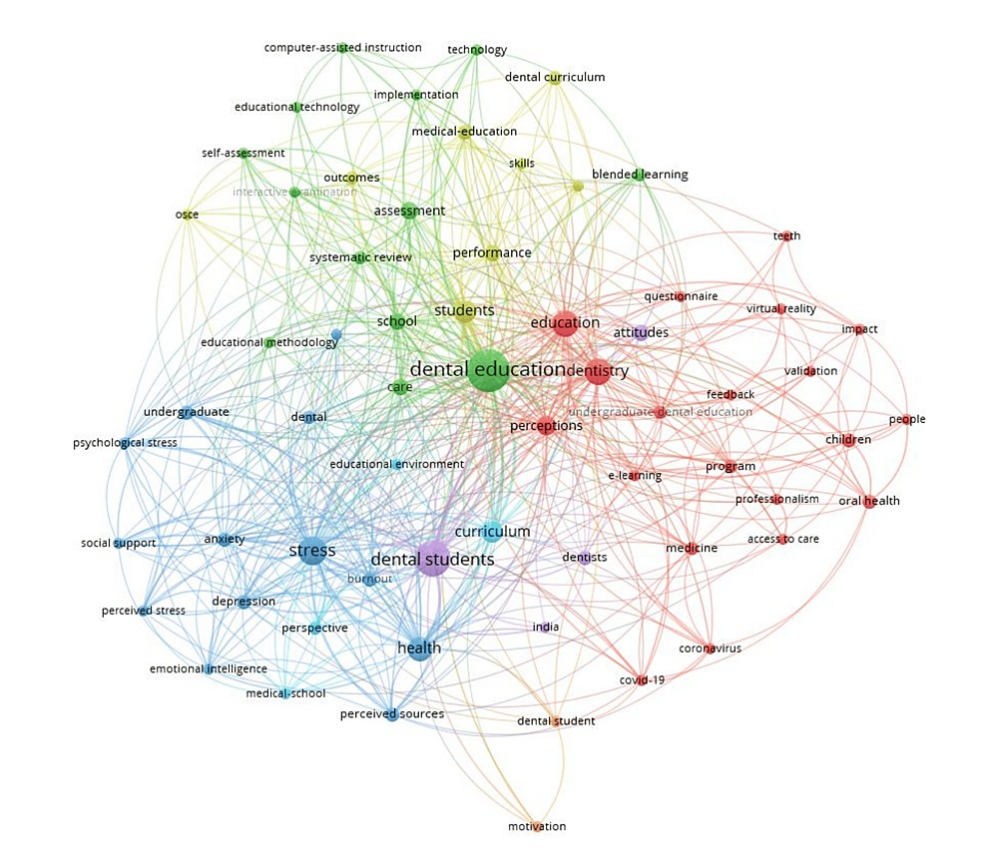
Keyword networks of the top 100 cited articles. Keyword cluster analysis revealed dental education (green) related to school, systematic review, assessment, blended learning, and self-assessment; stress (dark blue) related to anxiety, depression, undergraduate, health, burnout, and perceived stress; education (red) related to dentistry, perceptions, programs, professionalism, access to care, and virtual reality; students (yellow) related to performance, outcomes, skills, medical education, and dental curriculum; curriculum (light blue) related to perspective, educational environment, and medical school; dental student (orange) related to motivation; dental students (violet) connected to dentists and attitudes.

**Figure 4:**
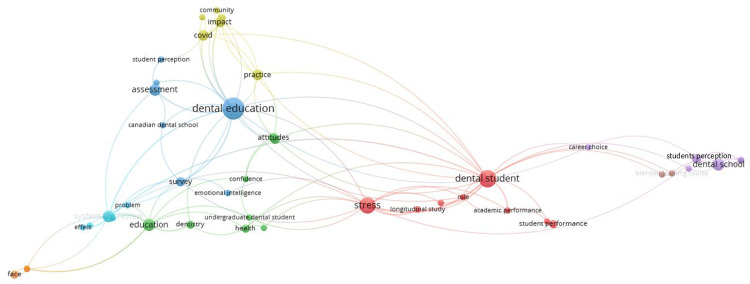
Title word networks of the top 100 cited articles. Title word analysis revealed dental education (dark blue) related to student perception, Canadian dental school, assessment, and survey; education (green) related to dentistry, health, confidence, and attitudes; practice (yellow) related to impact, community, practice, and COVID-19; dental student (red) related to stress, longitudinal study, academic performance, and student performance; dental school (violet) related to career choice and student perception; systematic review (light blue) related to effect and problem.

The keyword analysis of the top 100 cited articles showed a large focus on dental education linked to assessment and systematic review. A cluster linking stress, depression, anxiety, and health was observed. In addition, there was a cluster around education, dentistry, questionnaires, perceptions, virtual reality, and e-learning. Notably, clusters around words such as COVID-19 and coronavirus as well as computer-assisted instruction and educational technology were observed. The title word analysis of the top 100 cited articles showed a cluster around dental education and assessment; COVID-19 and practice; education and attitudes; dental students, stress, and academic performance; and dental school and career choice (Figures 3, 4).

The United States ranked highest in terms of publications for all periods analyzed. The other countries in the top six for all periods included England, Canada, Australia, and Saudi Arabia. Germany ranked fifth in the list between 2014 and 2018 and Brazil ranked third in the list between 2019 and 2023 (Table 3).

**Table 3:** Author-affiliated countries with the most publications during the years 2014-2018, 2019-2023, and 2014-2023.

2014–2018		2019–2023		2014–2023	
Country	Number of publications	Country	Number of publications	Country	Number of publications
United States	546	United States	712	United States	1,491
England	81	England	106	England	212
Canada	58	Brazil	83	Canada	160
Australia	41	Australia	77	Australia	123
Germany	39	Canada	76	Brazil	123
Saudi Arabia	30	Saudi Arabia	56	Saudi Arabia	87

Discussion

A bibliometric analysis allows the reader to get an overview of the areas of interest and publication trends in a specific topic or area of study [7,15]. This study analyzed trends in publications in the two journals focused on dental education: the Journal of Dental Education and the European Journal of Dental Education. A previous study performing a bibliometric analysis on endodontic education identified the lack of a comprehensive bibliometric analysis of dental education [15]. Only one previous bibliometric analysis was identified which examined dental education research between 2007 and 2013 [14]. Hence, this study aimed to fill that knowledge gap by examining publication trends in the last decade. It is interesting to note from the keyword and title word analysis that dental education and dental students were the most common occurrences in both periods as well as the top 100 cited articles. This is a positive finding as dental education and academia have faced significant challenges in recent times such as faculty shortages and faculty retention [19] in the last decade. Based on our analysis, it appears that despite these challenges, there is a strong emphasis in dental education research focused on dental students and dental education. In addition, interesting to note was the occurrence of stress which appeared in the top list of title words and keywords in the top 100 cited articles, further highlighting the challenges faced by the dental education fraternity. A previous bibliometric analysis of the top 100 cited articles also identified similar trends of keyword occurrences, including dental education and stress [5].

This study identified the United States to have the highest number of affiliated authors publishing in the two journals across the periods. The other countries in the top six list were consistent across the periods with only the order different among the different periods. The keyword network maps generated in this study identified the emergence of 3D printing, virtual reality, simulation, e-learning, and software during the period between 2019 and 2023, suggesting that the dental education research fraternity is keeping pace with the rapid technological advances in both dentistry and higher education [20,21]. It is also worth noting that the period between 2019 and 2023 had a significant cluster of keywords around COVID-19 that was linked to terms such as burnout, stress, anxiety, and depression, reflecting the trends of uncertainty observed worldwide during the pandemic and, in particular, by the dental fraternity.

This study is not without limitations. First, the search and the analysis were focused on the last decade. Although the two journals in this study have been publishing articles from earlier periods, the Web of Science database has only archives dating back to the late 2000s for these two journals. A second limitation identified is that the top 100 cited articles reported in this study were published within the period analyzed (2014-2023) and are not reflective of the 100 cited articles of all time. This is because the Web of Science does not allow the identification of earlier articles from the Journal of Dental Education and the European Journal of Dental Education and any bibliometric analysis of the overall top 100 cited articles in the two journals using the Web of Science database might be erroneous. Third, dental education research could be published in journals outside of the two journals research that were not identified in our search. However, it is a common trend in bibliometric analysis in the field of dentistry to identify key journals related to the field of interest due to the methodological feasibility of analyzing larger data and to permit the use of data mining tools to perform the analysis [22-24].

The article identified important trends in publications over the last decade in journals with a focus on dental education. Future research should focus on domain-specific research in dental education such as undergraduate and postgraduate dental education, as well as education articles pertaining to different dental specialties. Additionally, a comprehensive bibliometric analysis of all dental education research including articles published in journals other than the Journal of Dental Education and European Journal of Dental Education can be performed. Third, citation counts and the factors affecting them can be analyzed to provide more insights into the citation trends of articles focused on dental education.

## Conclusions

We performed a bibliometric analysis of title words and keywords from 2014 to 2023 in the Journal of Dental Education and the European Journal of Dental Education. Within the limitations of this study, we identified areas of interest in the field of dental education research over the last decade. These findings can help guide current and future research topics by identifying the current trends in journals focused on dental education research.
